# Mosquito-Host Interactions during and after an Outbreak of Equine Viral Encephalitis in Eastern Panama

**DOI:** 10.1371/journal.pone.0081788

**Published:** 2013-12-10

**Authors:** Wayra G. Navia-Gine, Jose R. Loaiza, Matthew J. Miller

**Affiliations:** 1 Smithsonian Tropical Research Institute, Panama City, Panama; 2 Centro de Biodiversidad y Descubrimiento de Drogas, Instituto de Investigaciones Científicas y Servicios de Alta Tecnología, Clayton, Panamá; 3 Programa Centroamericano de Maestría en Entomología, Vicerrectoría de Investigación y Postgrado, Universidad de Panamá, Panama City, República de Panamá; Universidade Federal do Rio de Janeiro, Brazil

## Abstract

Mosquito blood meals provide information about the feeding habits and host preference of potential arthropod-borne disease vectors. Although mosquito-borne diseases are ubiquitous in the Neotropics, few studies in this region have assessed patterns of mosquito-host interactions, especially during actual disease outbreaks. Based on collections made during and after an outbreak of equine viral encephalitis, we identified the source of 338 blood meals from 10 species of mosquitoes from Aruza Abajo, a location in Darien province in eastern Panama. A PCR based method targeting three distinct mitochondrial targets and subsequent DNA sequencing was used in an effort to delineate vector-host relationships. At Aruza Abajo, large domesticated mammals dominated the assemblage of mosquito blood meals while wild bird and mammal species represented only a small portion of the blood meal pool. Most mosquito species fed on a variety of hosts; foraging index analysis indicates that eight of nine mosquito species utilize hosts at similar proportions while a stochastic model suggests dietary overlap among species was greater than would be expected by chance. The results from our null-model analysis of mosquito diet overlap are consistent with the hypothesis that in landscapes where large domestic animals dominate the local biomass, many mosquito species show little host specificity, and feed upon hosts in proportion to their biomass, which may have implications for the role of livestocking patterns in vector-borne disease ecology.

## Introduction

Mosquito-host interactions are a critical factor in the dynamics and epidemiology of vector-borne diseases. In the case of arthropod-borne viral (arboviral) diseases, enzootic and bridge vectors feeding upon domesticated animals may cause spillover outbreaks in rural areas [1]. A combination of factors may determine a mosquito's biting choice, including innate genetic preference for certain hosts, host anti-biting behavior, and environmental factors [2]–[4]. Additionally, there is also growing evidence indicating that mosquito choices might be primarily driven by host availability [5], [6]. It is important to note that, fundamentally, the outcome of every mosquito-borne disease outbreak depends on which mosquitoes bite which vertebrates.

In May and June 2010, an equine viral encephalitis outbreak of mixed etiology occurred in eastern Panama (Darién province) involving both Venezuelan Equine Encephalitis Virus (VEEV) and Eastern Equine Encephalitis Virus (EEEV). Prior to the 2010 outbreak, the last reported case of EEEV in Panama was in 1986 [7], and VEEV was in 2004 [8]. The 2010 outbreak was novel among Panamanian outbreaks of equine encephalitis due to the relatively high number of human cases of EEEV, the first isolation of an enzootic strain of VEEV from horses, and a dual infection of EEEV and VEEV in one human [9]. In tropical America, both viruses are believed to be maintained in enzootic cycles by mosquitoes in the *Culex Melanoconion* subgenus [10], [11] with birds and mammals as the most likely vertebrate reservoirs respectively [1], [12], [13].

**Figure 1:**
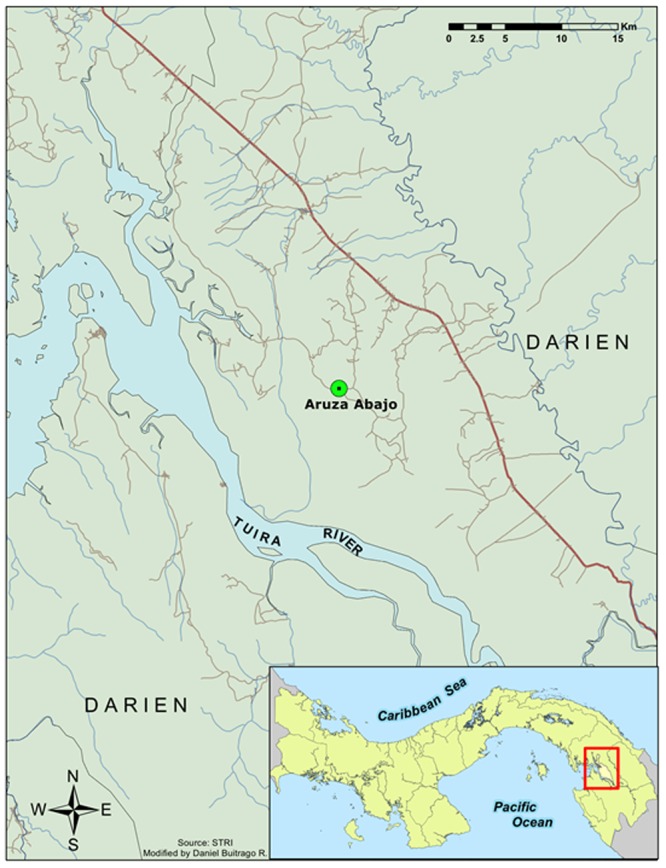
Map of Aruza Abajo study site in western Darien Province (Panama).

Opportunities to sample mosquito communities during an arbovirus outbreak are rare, especially in the tropics. Here we characterize the blood meals of a mosquito community sampled during and a few months after the 2010 encephalitis outbreak in Aruza Abajo, a rural community affected by the outbreak in western Darién (Figure 1). Aruza Abajo is part of the Rio Iglesias Corregimiento where two confirmed and one suspected cases of eastern equine encephalitis, and one suspected case of Venezuelan equine encephalitis, occurred among the 25 suspected or confirmed human cases occurring during the 2010 outbreak [9]. Our study goals were: (1) to document host-seeking mosquitoes at the site of an equine encephalitis outbreak in a peridomestic environment in Aruza Abajo; (2) to identify the vertebrate source of blood meals from these mosquitoes; and (3) to determine patterns of host use of some medically-important mosquito species.

We developed a comprehensive and reliable PCR method to analyze blood meals from individual blood-engorged mosquitoes. As a result, we identified 15 vertebrate hosts from a minimum of 10 mosquito species, being the first study in Panama to identify blood meals to the species level and allowing a preliminary assessment of the mosquito-host interactions at an epizootic/endemic focus of equine viral encephalitis.

## Materials and Methods

### Sampling location and collecting methodology

We were invited by the Panamanian Ministry of Agriculture (MIDA) to collect mosquitoes at locations in eastern Panama that suffered horse deaths suspected, and later confirmed, to be the result of equine viral encephalitis [9]. Our principle collections occurred in Aruza Abajo (8° 21.67′ N, 77° 56.44′ W), located approximately 185 kilometers southeast of Panama City (Figure 1). Aruza Abajo is a lowland humid area with uniform temperature throughout the year, ranging from 25 to 28°C. Humidity is generally high with an annual average rainfall of approximately 1800 mm [14], with most rainfall occurring between May and December. The landscape immediately surrounding Aruza Abajo is largely deforested with scattered patches of secondary forest intermingled with grassland, shrub vegetation and swampy areas. However, the site is located between the Filo de Tallo and Canglón Forest Reserves, each about 5 km away. Most of the people living in this locality are small-scale farmers who rear domestic animals at home and cultivate rice, plantains and maize among other focal crops. Cattle, horses, pigs, chickens, ducks and dogs, regularly used for consumption or to support manual labor at the farm, are the most common peridomestic animals.

Mosquitoes were collected using standard Centers for Disease Control and Prevention miniature light traps (John W. Hock Co., Gainesville, FL), as these were the most efficient means of sampling the mosquito community given the limited number of field personnel. The first collection round started on 18 June 2010 during the outbreak period whilst the second round started on 23 October 2010 during the post-outbreak phase. Both collections consisted of five consecutive nights of mosquito trapping. Six light traps operating continuously from sunset until next morning, were set up at 1.5 meters off the ground. Light traps were baited with one kilogram of solid carbon dioxide (e.g. dry ice, CO_2_) and were spaced 50–300 meters away from each other to avoid antagonism among them. Mosquitoes were frozen on dry ice to stop blood digestion and transported on dry ice to the Naos Molecular Biology and Evolution Laboratories of the Smithsonian Tropical Research Institute (STRI) where they were relocated to a −70°C ultracold freezer. Mosquitoes were identified to species level using a dissecting microscope, a chill table and morphological keys [15]–[17]. Blood-engorged females were then placed individually in labeled tubes at −70°C until DNA extraction. Field work was conducted with the following permits: IACUC, Smithsonian Tropical Research Institute, permit number 2011-0927-2014-03. The Panamanian Environmental Ministry approved mosquito collections in Panama under permit SE/A-12-08. All non-blood fed mosquitoes from these collections were exported to the Center for Disease Control - Ft. Collins for follow-up study of viral infection.

### DNA extraction and blood meal identification

DNA was isolated from intact engorged adult mosquitoes using a BioSprint 96 robot and associated DNA blood kit (QIAGEN, Valencia, CA). Each mosquito was placed in a well of a 96-well plate and crushed in tissue lysis buffer using a pestle followed by DNA isolation protocol from the manufacturer. Published vertebrate primers targeting cytochrome C oxidase I (COI), 16S ribosomal DNA (16S), and mammalian cytochrome-*b* (cyt-*b*) were used for this study, herein COI, 16S, and cyt-*b* primers, respectively (Table 1). Primary tests indicated that the COI primers were most efficient at vertebrate amplification; therefore all samples were initially screened with those primers. Samples that were negative in the first PCR were subsequently targeted using 16S and cyt-*b* primers. To assess the potential impact of primer set on blood meal identification, we attempted to amplify host DNA from 46 samples using all three primer sets. The amplification reactions contained 1X CoralLoad PCR buffer, 200 µM each dNTP, 0.2–0.6 µM of each primer and 0.2 units of Taq DNA polymerase (QIAGEN, Valencia, CA) and 1 µl of DNA template in a final volume of 15 µl. PCR conditions used with the COI primers consisted of 6 min at 96°C followed by 30 cycles of 40 sec at 94°C, 40 sec at 58°C and 1 min at 72°C, and a final extension step of 5 min at 75°C. PCR conditions used with the 16S primers consisted of 6 min at 96°C followed by 35 cycles of 40 sec at 94°C, 40 sec at 48°C and 1 min at 72°C, and a final extension step of 7 min at 75°C. The PCR conditions used with the cyt-*b* primers consisted of 6 min at 96°C followed by 35 cycles of 30 sec at 94°C, 30 sec at 54°C and 1 min at 72°C, and a final extension step of 4 min at 75°C. Positive and negative controls were included in each PCR reaction. The target PCR products were treated with ExoSAP-IT (USB Corporation, Cleveland, OH) to remove unincorporated dNTPs and PCR primers; where unspecific amplification was present, the PCR product was gel purified followed by GELase™ treatment (Epicentre Biotechnologies, Madison, WI). PCR products were cycle sequenced using BigDye Terminator v3.1 kit (Applied Biosystems, Foster City, CA) followed by Sephadex P-50 purification and sequencing using a 3130x1 Genetic Sequencer (Applied Biosystems, Foster City, CA). Sequences of 500 bp or more were entered into BLASTN (http://www.ncbi.nmln.nih.gov) or BOLD SYSTEMS v2.5 (http://www.barcodinglife.org) and the best match with identity of 95% or above was recorded. Sequences generated during this project are available on GenBank (accession numbers: KF799977-KF799997).

**Table 1:** PCR primers used to amplify mitochondrial DNA of mosquito blood meals.

Primer Name	Sequence (5′ – 3′)	Target	Reference
COI Forward	TTCTCCAACCACAAAGACATTGGCAC	Cytochrome Oxidase I	[46]
COI Reverse	ACTTCTGGGTGGCCAAAGAATCAGAA	Cytochrome Oxidase I	[47]
16Sar	CGCCTGTTTATCAAAAACAT	16S	[48]
16Sbr	CCGGTCTGAACTCAGATCACGT	16S	[48]
Mammalian-F	CGAAGCTTGATATGAAAAACCATCGTTG	Cytochrome-*b*	[27]
Mammalian-R	TGTAGTTRTCWGGGTCHCCTA	Cytochrome-*b*	[27]

### Mosquito host preference and avoidance

For each species of mosquito we calculated the Foraging Index (*FI*) [18], [19], which is the ratio of observed blood meals from a particular host for a particular mosquito species compared to the overall proportion of a given host's blood meal across all hosts. Values near 1.0 indicate that the particular mosquito is feeding proportionally compared to all mosquitoes, whereas values above 1.0 show a preference for that host and values below 1.0 show avoidance of that host. Statistical significance of *FI* was computed by a two-tailed exact binomial test for goodness of fit. We performed two analyses, one on specific hosts: pig, cow, horse, dog, human, other mammals, chickens, and other birds; and a second on all birds vs. all mammals. Significance was evaluated after applying Holms-Bonferroni correction for multiple tests to constrain the familywise error rate (e.g. overall *P*-value) to 0.05.

### Null models to test for random or structured mosquito feeding patterns

Several methods to analyze blood-feeding patterns in mosquitoes have been developed previously, including the calculation of host utilization rate, forage ratio, and feeding index. However, most of these analyses involve gathering data on host relative abundance, which can be very difficult to obtain, especially in tropical regions. Therefore, we chose a null-model to test whether mosquito feeding habits during and after the outbreak were random or structured [5]. This null-model analysis generates a C-score for a set of captured mosquitoes and their vertebrate host blood meals. Intermediate C-score values indicate acceptance of the null model: i.e. random feeding pattern. Alternatively, high C-score values indicate a segregated pattern, meaning that mosquito species have host-specific preferences, while low C-score indicate an aggregated pattern, characteristic of mosquito communities that feed upon the same host species. We measured the significance of the C-score of the outbreak and post-outbreak samples as well as the pooled samples using Ecosim 7.0 [5], [20].

We generated a quantitative interaction network using the bipartite package [21] following authors' instructions in the R statistical package [22]. Importantly, compared to other interaction networks, the bipartite approach generates quantitative networks that show the relative numerical importance of the various interactions in the network.

We compared the number of blood meals from the various sources (domestic animals species, wild bird species, wild mammal species, and humans) to estimates of the biomass for these sources in the local landscape. Biomass estimates for domestic animals were derived from observational point counts of peridomestic and agricultural sites around Aruza Abajo. Estimates of wild mammal and bird biomass were derived from the literature, and human biomass estimates were based on the 2010 population census for Aruza and surrounding areas [23]. A full description of methods used to generate biomass estimates can be found in Methods S1. Furthermore, we estimated human population and domestic animal population growth using recent household and agricultural census data [23], [24]. To evaluate the relationship between biomass and mosquito-biting patterns, the square-root transformed relative abundance of vertebrate host species in our blood meal samples was regressed against the log_10_-transformed host species biomass.

## Results

### Mosquito species composition

In June 2010, at the highpoint of the 2010 outbreak, we collected 3,270 mosquitoes in Aruza Abajo (Darién), representing at least 20 species and 10 genera (Table 2). The most abundant species was *Coquillettidia venezuelensis* (50.6%) followed by *Culex nigripalpus* (17.0%) and *Psorophora cingulata* (10.2%), the rest of the *Culex Culex* subgenus (13.2%), and members of the *Culex* (*Melanoconion*) subgenus (7.2%, which were dominated by *Cx. Mel. pedroi*: 1.7%). Comparatively, in October 2010, when no cases of encephalitis were reported and the outbreak was presumably over, we returned to Aruza Abajo and collected 4,228 mosquitoes, which represented at least 17 species and 7 genera (Table 2). As in the June mosquito collection, the most abundant species in the October samples was *Cq. venezuelensis* (23.6%) followed by *Cx. nigripalpus* (20.9%), *Ps. cingulata* (17.5%), *Mansonia indubitans* (9.4%), the *Cx*. (*Mel*.) subgenus (8.5%, which were dominated by *Cx*. (*Mel.*) *pedroi*: 3.5%), the remaining member of the *Cx.* (*Cux.*) subgenus (including many mosquitoes that could not be identified to species, 6.7%), *Uranotaenia lowii* (3.6%), *Ur. apicalis* (2.7%), *Anopheles triannulatus* (2.3%), *Aedeomyia squamipennis* (2.2%), *Ma. titillans* (1.6%).

**Table 2:** Proportion of mosquitoes collected per species before and after the 2010 encephalitis outbreak in Aruza Abajo, Darien, Panama.

Species	Outbreak		Post-outbreak	
	*N*	%	*N*	%
***Coquillettidia venezuelensis***	**1655**	**50.6%**	**996**	**23.6%**
***Culex*** ** (** ***Culex*** **)**	**986**	**30.2%**	**1171**	**27.7%**
*– Culex* (*Cux.*) *nigripalpus*	555	17.0%	884	20.9%
*– Culex* (*Cux.*) *interrogator*	56	1.7%	21	0.5%
*– Culex* (*Cux.*) *declarator*	5	0.2%	65	1.5%
*– Culex* (*Cux.*) *coronator*	28	0.9%	31	0.7%
*– Culex* (*Cux.*) *mollis*	8	0.2%	–	–
*– Culex* (*Cux.*) *quinquefasciatus*	3	0.1%	–	–
*– Culex* (*Culex*) *sp.*	331	10.1%	170	4.0%
***Psorophora cingulata***	**333**	**10.2%**	**738**	**17.5%**
***Culex*** ** (** ***Melanoconion*** **)**	**235**	**7.2%**	**361**	**8.5%**
*– Culex* (*Mel.*) *pedroi*	55	**1.7%**	146	3.5%
*– Culex* (*Mel.*) *taeniopus*	8	**0.2%**	–	–
*– Culex* (*Melanoconion*) *sp.*	172	**5.3%**	215	5.1%
***Mansonia indubitans***	**3**	**0.1%**	**396**	**9.4%**
***Uranotaenia***	**4**	**0.1%**	**306**	**7.2%**
*Uranotaenia lowi*	–	–	151	3.6%
*Uranotaenia apicalis*	4	0.1%	115	2.7%
*Uranotaenia pulcherrima*	–	–	13	0.3%
*Uranotaenia histera*	–	–	10	0.2%
*Uranotaenia sp.*	–	–	17	0.4%
***Anopheles***	**8**	**0.2%**	**101**	**2.4%**
*– Anopheles punctimacula*	–	–	4	0.1%
*– Anopheles albimanus*	5	0.2%	–	–
*– Anopheles triannulatus*	–	–	96	2.3%
*– Anopheles apicimacula*	–	–	1	0.0%
*– Anopheles sp.*	3	0.1%	–	–
*– Anopheles* (*Nyssorynchus*) *sp.*	1	0.0%	–	–
***Aedeomyia squamipennis***	**2**	**0.1%**	**93**	**2.2%**
***Mansonia titillans***	**12**	**0.4%**	**66**	**1.6%**
***Psorophora ferox***	**16**	**0.5%**	–	–
***Psorophora albipes***	**7**	**0.2%**	–	–
***Aedes fulvus***	**5**	**0.2%**	–	–
***Psorophora ciliata***	**3**	**0.1%**	–	–
**Total**	**3270**		**4228**	
				

Sampling effort was the same during the outbreak and in the post-outbreak period, representing a total of 30 trap nights per period.

### Molecular identification of mosquito blood meals

**Figure 2:**
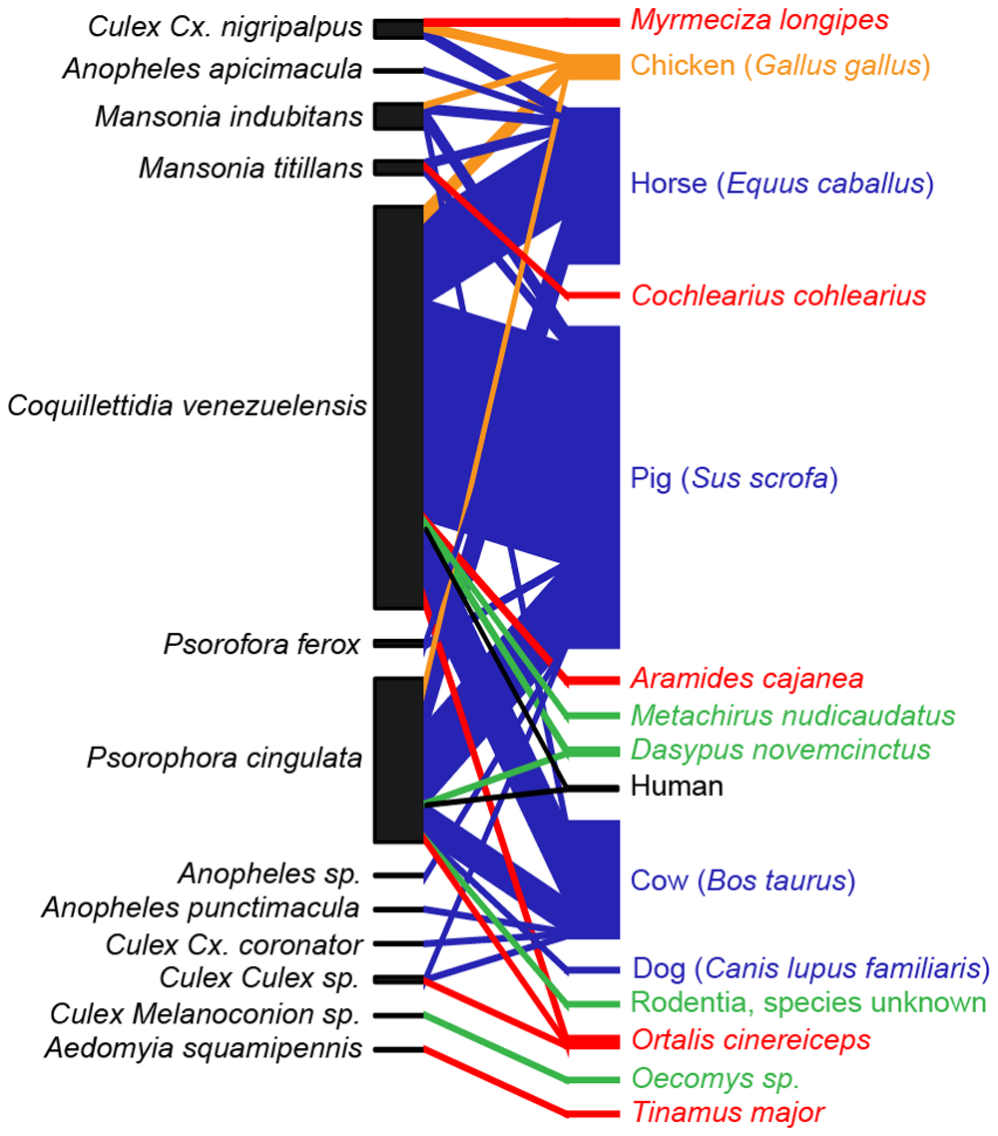
Host-vector quantitative interaction network based on the molecular identification of 338 mosquito blood meals from Aruza Abajo, Panama. Red lines represent interactions involving wild birds, green lines: wild mammals, orange lines: chickens, blue lines: domestic mammals, and black lines: humans.

We identified the source of 338 blood meals out of a total sample of 430 blood-engorged mosquitoes collected in Darién in June and October 2010, for a total efficiency of 78.6% using primers targeting the mitochondrial COI, 16S, and cyt*-b* regions (Table 1). The identified hosts included 9 mammals and 6 bird species, representing 5 domesticated animal species, 9 wild species, and humans (Figure 2). Primers targeting COI and 16S genes amplified DNA from 8 species each, however the 16S primers often amplified mosquito DNA instead of the vertebrate blood meal. The primers targeting mammalian cytochrome-*b* amplified only 5 vertebrate species. In total, 74.3% of the blood meals were identified using COI primers and 15.7% and 10% using mammalian cyt-*b* and 16S primers, respectively. Our approach usually identified the same blood meal independent of primer pair used based on a subset of 46 samples tested with multiple primer pairs, 38 (82.6%) recovered the same vertebrate host, while five samples (10.9%) recovered one vertebrate host on the forward sequence and another vertebrate host with the reverse primer generated from the same COI amplification. Only three samples (6.5%) recovered one host with one primer pair and a different host with a second primer pair. We assume that in both cases, this is the result of multiple blood meals in the sample, and as such included both results in all subsequent analyses. During the outbreak period the most common vertebrate host among all mosquito species were domestic animals: pig (*Sus scrofa*, 58.5%), horse (*Equus caballus*, 21.5%), cow (*Bos taurus*, 13.6%), and chicken (*Gallus gallus*, 3%). After the outbreak, the most common hosts were the same: cow (33.8%), horse (29.9%), pig (18.2%), and chicken (3.9%). Wild animals were less common in both samples; after the outbreak we also recovered two human blood meals, one from *Cq. venezuelensis* and another from *Ps. cingulata*.

### Mosquito-host interactions

Host richness per species of mosquito varied between one and nine, out of a total pool of 15 vertebrate species recovered. Blood meals from the two most commonly blood engorged mosquitoes captured in this study were dominated by mammals. *Cq. venezuelensis* fed mostly on domestic mammals (93%), with only limited feeding on domestic birds (3%), wild birds (2%), wild mammals (1%) and humans (1%). Likewise, blood meals for *Ps. cingulata*, were dominated by domestic mammals (94%), with wild mammals (3%), wild birds (1%), domestic birds (1%), and humans (1%) representing a small portion of the diet (Tables 3 & 4).

**Table 3:** Counts of blood meals identified using DNA sequencing from mosquitoes collected at Aruza Abajo during an outbreak of equine encephalitis.

Blood meal	*Coquillettidia venezuelensis*	*Psorophora cingulata*	*Mansonia titillans*	*Culex Cx. nigripalpus*	*Psorophora ferox*	*Culex sp.*	*Anopheles sp.*	Total
Pig (*Sus scrofa*)	110	37	3	0	2	1	1	**154**
Horse (*Equus caballus*)	41	9	3	1	1	0	0	**55**
Cow *(Bos taurus)*	30	4	0	0	0	0	0	**34**
Nine-banded Armadillo (*Dasypus novemcinctus*)	1	0	0	0	0	0	0	**1**
Chicken (*Gallus gallus*)	6	1	0	1	1	0	0	**9**
Gray-headed Chachalaca (*Ortalis cinereiceps*)	3	1	0	0	0	1	0	**5**
Gray-necked Wood-Rail (*Aramides cajanea*)	2	0	0	0	0	0	0	**2**
Boat-billed Heron (*Cochlearius cochlearius*)	0	0	1	0	0	0	0	**1**
**Total**	**193**	**52**	**7**	**2**	**4**	**2**	**1**	**261**

**Table 4:** Counts of blood meals identified using DNA sequencing from mosquitoes collected at Aruza Abajo after an outbreak of equine encephalitis.

Blood meal	*Coquillettidia*	*Psorophora*	*Mansonia*	*Culex Cx.*	*Aedomyia*	*Culex*	*Culex Cx.*	*Culex*	*Anopheles*	*Anopheles*	Total
	*venezuelensis*	*cingulata*	*indubitans*	*nigripalpus*	*squamipennis*	*Cx sp.*	*coronator*	*Melanoconion sp.*	*apicimacula*	*punctimacula*	
Pig (*Sus scrofa*)	5	3	6	0	0	0	0	0	0	0	**14**
Horse (*Equus caballus*)	3	13	3	3	0	0	0	0	1	0	**22**
Cow *(Bos taurus)*	8	12	3	0	0	1	1	0	0	1	**25**
Dog (*Canis lupus familiaris*)	0	1	0	0	0	0	0	0	0	0	**1**
Human (*Homo sapiens*)	1	1	0	0	0	0	0	0	0	0	**2**
Brown Four-eyed Opossum (*Metachirus nudicaudatus*)	1	0	0	0	0	0	0	0	0	0	**1**
Nine-banded Armadillo (*Dasypus novemcinctus*)	1	1	0	0	0	0	0	0	0	0	**2**
Arboreal Rice Rat (*Oecomys sp.)*	0	0	0	0	0	0	0	1	0	0	**1**
Rodentia, undetermined	0	1	0	0	0	0	0	0	0	0	**1**
Chicken (*Gallus gallus*)	0	0	1	2	0	0	0	0	0	0	**3**
White-bellied Antbird (*Myrmeciza longipes*)	0	0	0	2	0	0	0	0	0	0	**2**
Great Tinamou (*Tinamus major*)	0	0	0	0	1	0	0	0	0	0	**1**
**Total**	**19**	**32**	**13**	**7**	**1**	**1**	**1**	**1**	**1**	**1**	**75**

However, results from a statistical test of *FI* for these taxa show that these foraging patterns are consistent with the overall foraging patterns for all blood-engorged mosquito species analyzed. Of the nine blood-fed species examined, only *Cx.* (*Cux*). *nigripalpus* showed signs of diet specificity that differed significantly from the overall pattern –namely preferring birds to the exclusion of mammals more than could be expected by chance (*FI* = 8.4; two-tailed exact binomial test: *P* = 0.00015, which remains significant after applying a Holm-Bonferroni correction resulting in a critical value of *P* = 0.00556; see Table S1). Likewise, when we evaluate species-specific deviations from 1.0 foraging indices, we find five cases where foraging indices resulted in an individual *P*-value for a given host less than 0.05, four involving *Cx.* (*Cux.*) *nigripalpus*: avoidance of pigs (*FI* = 0.0), preference of horses (*FI* = 1.9), preference for chickens (*FI* = 9.3), and preference of other birds (*FI* = 6.8), and a fifth case, *Aed. squamipennis*, which showed a preference for other birds (*FI* = 30.6). However, after controlling for a familywise error rate of 0.05 via Holm-Bonferroni correction, we find that only the preference for chickens in *Cx.* (*Cux.*) *nigripalpus* is significant. In general, the trend throughout our dataset, where 68 of 72 species-specific *FI*s did not differ from 1.0, and where 8 of 9 vertebrate class-based tests likewise did not differ from 1.0, provides evidence that many of the mosquito species in Aruza Abajo, except for an apparent preference for bird hosts in *Cx.* (*Cux.*) *nigripalpus*, did not show innate preferences for particular vertebrate hosts. However, we recognize that it is possible that some significant deviations of *FI* scores from 1.0 were hampered by low sample size.

This finding agrees with the results of our null model test which found aggregated mosquito feeding patterns, meaning that diet overlap among mosquito species was greater than could be expected by chance alone (C-score: 1.85, *p*<0.01). Finally, an interaction network describing the relationship between mosquito species and vertebrate hosts presents a visual confirmation that the dominant host-mosquito interactions were between the most abundant mosquito species and large domesticated mammals, but that most mosquito species feed on both birds and mammals, and both wild and domestic animals (Figure 2). Collectively across the various analytical approaches used in this study we find little evidence for diet variation among the blood-fed mosquitoes apart from an apparent preference for birds for *Cx.* (*Cux.*) *nigripalpus*.

### Mosquito diets and estimates of wildlife and livestock biomass

**Figure 3:**
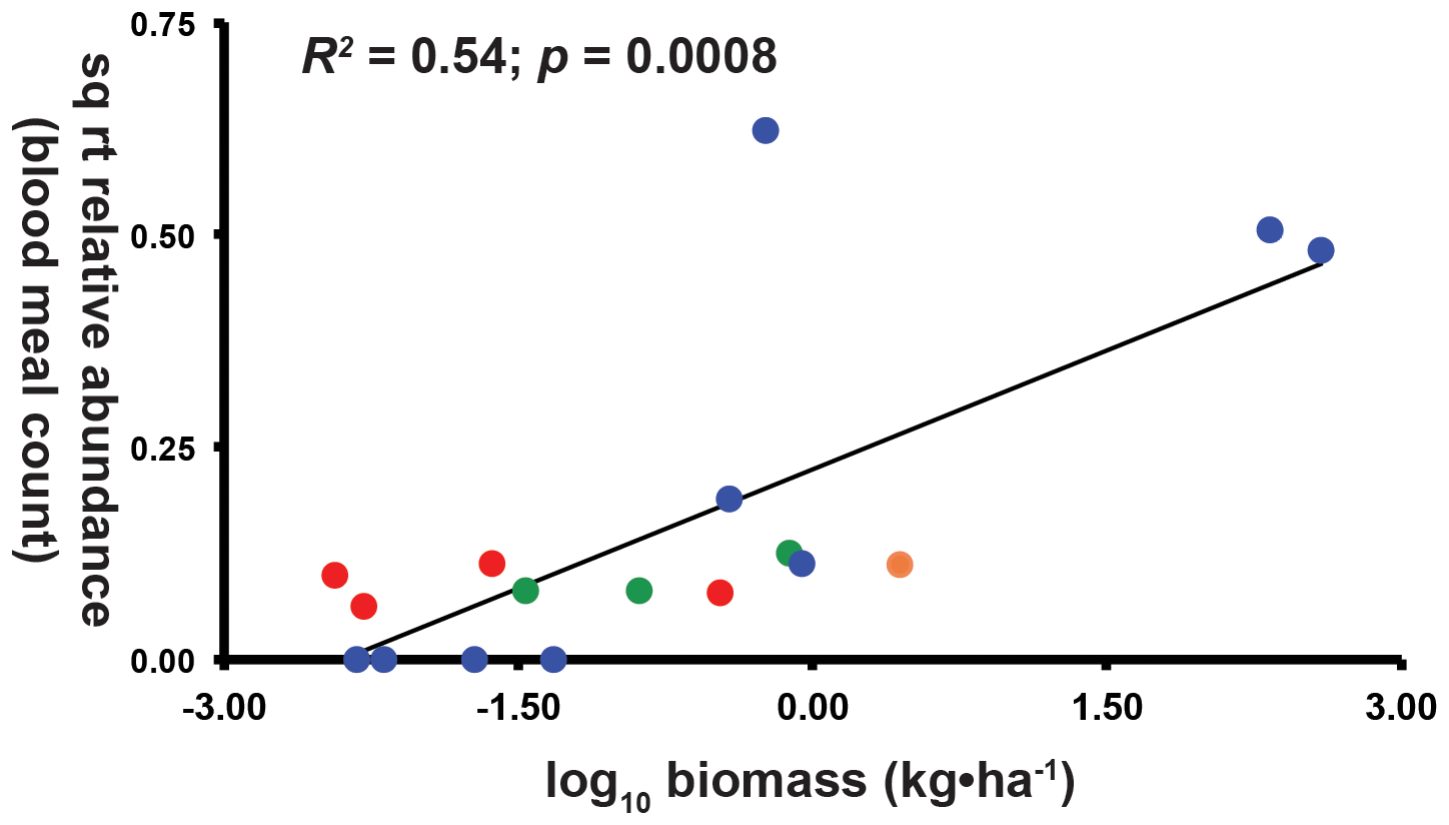
Relative abundance of vertebrate blood meals as a function of estimated host biomass. Red circles represent wild birds, green circles: wild mammals, orange circle: chickens, blue circles: domestic mammals, humans: black circle. Wild animals have both low biomass and blood meal relative abundance while humans and domestic animals have both high biomass and proportion of the observed blood meals. Estimated host biomass is log-transformed, while blood meal relative abundance is square-root transformed, with data representing biomass for taxa across the matrix of agricultural and forest habitats, see Methods S1 for details on methodology.

Square-root transformed relative abundances of host blood meals in our sample were significantly correlated with log_10_-transformed estimates of host biomass in the Aruza Abajo area (Figure 3: least-squares linear regression: *R^2^* = 0.54; *p* = 0.0008). Importantly, wild birds and mammals had low representation among our identified blood meals and low estimated biomass in the Aruza Abajo region while species of domestic animals (pigs, cows, and horses) had high estimated biomass and relative abundance among the vertebrate blood meals recovered in our study. Other domestic animals had moderate and low biomass estimates, which correlated with moderate and low frequency of blood meal recovery in our study. Humans had both moderate relative abundance among recovered blood meals and moderate estimated biomass.

## Discussion

Our molecular tool kit of three primer sets targeting three different vertebrate mitochondrial regions was relatively efficient at identifying the vertebrate host of mosquito blood meals (78.6% of blood meals from engorged mosquitoes were able to be identified). Furthermore, there is little evidence that different primer sets resulted in different blood meal determination, as might have been expected due to preferential amplification of primers in the case of multiple blood meals. However, our efficiency did not reach 100%, and so additional work to generate a set of universal primers for mosquito blood meals needs to be developed. However this is hampered by the difficulty of finding priming regions that are conservative enough to amplify across various classes of vertebrates; yet do not also amplify DNA from the invertebrate vector. Prior to this paper, most studies have used primers that focus on a particular class of potential vertebrate hosts [25], [26], only identify host to higher taxonomic level [27] or were based in the temperate region where both vector and host species richness is considerably poorer than in the Neotropics [28]. One exception is Muturi et al [29] who used a similar multiple primer set approach to identify blood meals of Tsetse flies in eastern Africa. Nonetheless, it is worth pointing out that PCR-based identification can introduce potential biases in bloodmeal studies: the failure to successfully amplify 20% of our samples may be due to host-species specific primer failures, and there is a potential that preferential amplification of one blood meal source over another may mask multiple feeding events. However, alternatives such as ELISA-based analyses are not practical in tropical settings where the potential host species pool can be over 50 species [29].

Three mosquito species dominated our samples during and after the encephalitis outbreak *Cq. venezuelensis*, *Cx.* (*Cux.*) *nigripalpus* and *Ps. cingulata*. A previous study [30] found that *Cq. venezuelensis* fed primarily upon mammalian hosts, and only occasionally on wild birds in central Panama. This outcome agrees with our findings since most blood meals from this species were identified to be from domestic pigs, cows and horses, but we found blood from chickens and two non-passerine species of wild birds associated with tropical forests in *Cq. venezuelensis* blood meals. *Cq. venezuelensis* has been implicated as a vector of VEEV based on viral isolations in northeastern South America [31]. *Cx.* (*Cux.*) *nigripalpus* comprised 17% of the outbreak mosquito sample and 21% of the post-outbreak sample. An additional 10% and 4% of these samples respectively could only be identified to the *Cx.* (*Cux.*) subgenus; however, given that 87% of the identified members of subgenus *Cx.* (*Cux.*) were *nigripalpus* among fully-identified members of this subgenus in our samples, it appears that the actual relative abundance of *nigripalpus* may be as high as 25% (see Table 2). Despite a high relative abundance of this taxon in our samples, relatively few were blood engorged. Nonetheless, we recovered blood meals from wild birds, chickens, and horses in those samples. Lineage III of EEV was isolated from a pool of *Cx.* (*Cux.*) *nigripalpus* collected in Trinidad in 1959 [32] and has also been shown to carry VEEV [33], [34]. It should be noted that human blood meals were only recovered from *Ps. cingulata* and *Cq. venezuelensis*, but as mentioned above this pattern may be due to sample size effects rather than innate diet preferences. Earlier work suggests that two other *Psorophora* species, *Ps. albigenu* and *Ps. ferox* are both bridge vectors of South American strains of EEEV (Lineages II and III), but that *Ps. cingulata* likely cannot transmit the virus due to a salivary gland barrier [35].

Alternatively, some mosquito species suggested by other studies as having a potential central role in Neotropical equine encephalitis transmission were absent or at low abundance in our Aruza Abajo samples. *Aedes taeniorhynchus* has been suggested as a potential bridge vector for North American EEEV lineages [35], [36], and one previous study suggested that this species might transmit EEEV in central Panama due to its high abundance and preferences for horses [30]. We collected no specimens of this species in 2010 at Aruza Abajo. Likewise, *Cx*. (*Mel*.) *pedroi* is considered to be the primary vector of EEEV in the Peruvian Amazon [13], and is also a primary vector of VEEV in northern Colombia [37]. In Aruza Abajo, *Cx*. (*Mel*.) *pedroi* was collected in low numbers (about 4% and 3% of the total samples, respectively, after applying the same correction employed above to *nigripalpus*). These low abundances might argue against a principle role for either *Ae. taeniorhynchus* or *Cx*. (*Mel*.) *pedroi* in the 2010 equine encephalitis outbreak in eastern Panama; but an alternative hypothesis, that our exclusive use of CDC light traps under-sampled these species, cannot be rejected. It should be pointed out that our characterization of the mosquito species assemblage is based on just two short sampling periods, and additional sampling using alternative mosquito traps (e.g., resting boxes), is likely to add additional species as well as more individuals of *Cx*. (*Mel*.) *pedroi*.

Collectively, our data reinforce the notion that mosquito species have broad diets [5], [6], [38], [39]. The results from our null model analysis are in agreement with most recent literature on vector-host interactions, suggesting that host abundance determine mosquito-feeding choices [5], [6]; specifically, the log-linear relationship between the number mosquitoes collected for a given species and the blood meal species richness for that species (Figure 3), is incompatible with previous notions that most mosquitoes have narrow feeding profiles (e.g. [30]). Instead, our results suggest that the pattern of extremely varied diets for abundant species such as *Cq. venezuelensis* (9 host species) and *Ps. cingulata* (9 host species) would be also found in the remaining, less abundant mosquito species had we sampled more blood-fed females. However, it should be noted that our sample size of engorged mosquitoes was quite low for several species, and it is likely that many mosquito–host interactions were not recovered in our sampling. For example, no human blood meal was recovered during the outbreak.

Until quite recently, most studies of mosquito blood meals have reinforced the notion of mosquito diet specificity. For example, a recent study in northeastern USA [40] found that 100% of *Cx. restuans* and 93% of *Cx. pipiens* blood meals came from birds, with more than 60% of these referring to just three wild birds species. An Australian study found that 75% of avian blood meals identified from one species of *Culex* in one collection location referred to only three bird species, while another *Culex* species collected at three sites had 75% of its avian blood meals assignable to dabbling ducks [41]. A study from southeastern Brazil found apparently high diet specificity in five mosquito species using serological tests [42]. However, our finding of broad diets among Panamanian mosquitoes agrees with Tempelis and Galindo [39], who also found varied a tendency for mosquito diets mixed between mammal, birds, and herptofauna, albeit in different species of mosquitoes in Panama than the ones in our study. It is unclear what is responsible for the qualitative difference in mosquito diet breadth between these studies and ours, but we do note that the above cited studies come from extra-tropical regions or use non-molecular assays and we believe that additional studies of mosquito diet breadth using DNA barcoding approaches from tropical regions are warranted.

Although feeding patterns among Aruza Abajo mosquitoes are broad and varied, they are not random. Rather, among mosquito species, mosquito-host interactions are aggregated, whereby the degree of diet overlap among mosquito species is greater than would be expected were feeding patterns random. Critically, the aggregation is towards three species of domestic mammals (pigs, cows, and horses) that dominate mosquito blood meals from Aruza Abajo. The over-aggregation of mosquito feeding on domestic animals can be explained by the dominance of these domestic animals biomass relative to estimated values of wild bird and mammal and other domestic animal biomass for the area (Figure 3). To wit, mosquitoes at Aruza Abajo are feeding on domestic animals in proportion to these animals' relative biomass on the local landscape [6], and suggests that researchers should consider changes in livestock patterns along with deforestation [43], [44], and the loss of biodiversity [45] when evaluating the consequences of land use change on emerging diseases in tropical regions.

## Supporting Information

Table S1 **Foraging indices of mosquitoes collected during and after an encephalitis outbreak, based on molecular blood meal identifications.**
(XLSX)

Methods S1 **Methodology underlying vertebrate biomass estimates in the area surrounding Aruza Abajo.**
(DOCX)
