# Supplementary material for: Mosquito-Host Interactions during and after an Outbreak of Equine Viral Encephalitis in Eastern Panama
Source: PLoS One. 2013 Dec 10;8(12):e81788. doi: 10.1371/journal.pone.0081788 (PMC3858258; doi:10.1371/journal.pone.0081788)
Supplement: Methods S1 — Methodology underlying vertebrate biomass estimates in the area surrounding Aruza Abajo. (DOCX) [file pone.0081788.s002.docx]

**Methods S1:** **Methodology underlying vertebrate biomass estimates in the area surrounding Aruza Abajo.**

Biomass estimates for domestic animals came from point counts conducted between 20 March and 10 April 2011 in and around Aruza Abajo. A total of 20 points were surveyed. 10 points were centered on the area immediately surrounding a family dwelling, and 10 points were located in an agricultural area, usually pasturelands, but occasionally crops. Each point had a radius of 50 meters, and all domestic occurring in the point space during a five-minute observation period. Each point was surveyed eight times in a three-day period.

Using GIS software, we estimated the total land area covered by the points to be roughly 3000 hectares, and included four named places in the 2010 Panama national census [32]: Aruza Abajo, Los Mellos, Agua Caliente, Quebrada Seca. That census records 48 households and 168 residents for this area.

Most domestic animals (only horses and cows) were never counted in the agricultural points, so we estimated these species’ biomass only from the family dwelling point counts. For each of the 10 family dwelling points, the maximum number of animals from each species from the eight count replicates was used as the species count for that dwelling. This was done because it is likely that values less than the minimum were due to animals being obscured from view, either in the house or in a pen. We then averaged the maximum value for all 10 dwellings, and used that as the estimate of per household abundance for the study area. We multiplied this average value by 48 (the total number of households in the area) and divided by 3000 hectares to obtain density hectare^-1^ estimates for pigs, chickens and dogs. We used the following mass estimates to convert density estimates to biomass hectare^-1^ estimates:

pigs: 50 kg

chickens: 1.2 kg

dogs: 25 kg

cats (*Felis catus*): 2 kg

domesticated muscovy ducks (*Cairina moschata*): 5 kg

helmeted guineafowl (*Numida meleagris*): 4 kg

blue and yellow macaws (*Ara ararauna*) : 1.2 kg

Cows and horses were equally observed immediately around dwellings and in the agricultural points, so we estimated their density directly from the circle area, rather than by household, but instead of taking the maximum value for a count, we used the averaged observed for all eight replicates as the value for each circle. This is because these animals tended to wander in and out of the count area, and grazed in an area considerably larger than the point circle. We took the average animal count for all 20 circles and converted this to density hectare^-1^. Density estimates were converted to biomass hectare^-1^ estimates assuming the following masses:

cow (most cattle are sold at 1 year): 250 kg

horse: 300 kg

Estimates of wild bird and mammal densities were taken from published ecological studies. Bird data came from Robinson [50], who reported on density and body mass estimates from two previous studies [51,52] undertaken in humid forest in Panama. We took the average of the two density estimates to obtain and estimate of biomass hectare^-1^. Estimates of wild mammal biomass density were obtained from [53] which provided average values of mammal density and mass for the Neotropics. It should be noted that the wild animal biomasses may be overestimated in the Aruza Abajo area to the extent that they are derived from intact forest surveys given the highly fragmented nature of the Aruza Abajo landscape.

Relative abundance of blood meals for each vertebrate species were calculated from the outbreak (N = 261) and post-outbreak (N = 77) samples, and the average relative abundance for each vertebrate species was compared by linear regression to the natural logarithim of each species’ Aruza Abajo biomass estimate.

49. Robinson WD (2011) Changes in abundance of birds in a Neotropical forest fragment

over 25 years: a review. Animal Biodiversity and Conservation 24: 51-65.

50. Wills EO (1980) Ecological roles of migratory and resident birds on Barro Colorado Island, Panama. In: Migrant birds in the Neotropics: ecology, behavior, distribution and conservation: 205–225 (Keast, A & Morton, ES eds.). Smithsonian, Washington,

DC, USA.

51. Robinson WD (1999) Long–term changes in the avifauna of a tropical forest isolate, Barro Colorado Island, Panama. Conservation Biology, 13: 85-97.

52. Robinson, JG, Redford, KH (1986) Body size, diet, and population density of Neotropical forest mammals. Amer Nat. 128:665-680.
